# Enforcing the Canadian Prohibition of Overcrowding Livestock in Transit Without Resorting to Science

**DOI:** 10.3390/ani15172612

**Published:** 2025-09-05

**Authors:** Terry L. Whiting

**Affiliations:** Independent Researcher, 191 Lawndale Ave, Winnipeg, MB R2H 1T4, Canada; terrywhiting9@gmail.com; Tel.: +1-204-794-7875

**Keywords:** humane transport, animal welfare, overcrowding, soft law, symbolic law, outcome-based regulation, animal law, street level bureaucrat

## Abstract

Administrative law is about articulating norms and promoting the adoption and enforcement of human behaviour practices in areas where individual choice conflicts with the public good. Law must fairly describe to the citizenry what is allowed and what is conditionally restricted by administrative law and what is prohibited in criminal law. The conditional use of animals in industrial production of food and fibre for human consumption is allowed (non-criminal), but the conditions which are considered acceptable by society are changing. Humane transportation of livestock is a public concern and the temptation of transporters to load as many animals as possible is an easily understood risk to animal welfare. Livestock hauling has been regulated in Canada since 1857 and is currently regulated by the Health of Animals (Act) Regulations Part XII and enforced nationally by the Canadian Food Inspection Agency. After over 30 years of challenging industry consultation, a major revision to the humane transport provisions was proclaimed in 2020. Surprisingly, the revision failed to articulate numerical thresholds that clearly indicate an overcrowding violation. This paper reviews 20 cases from 2004 to 2024 where transporters appealed a penalty for overcrowding livestock transported by land. The paper describes the decision to penalize transporters where there is no bright line definition of an offence. The paper suggests both regulator and regulated choose to work in an intentionally inefficient institutional arrangement, preferring opacity to clarity in what constitutes a violation in law, because “that’s the way we like it”.

## Simple Summary

In Canada, animal protection law, duty to care for animals, is primarily the constitutional responsibility of the provincial governments (Administrative Law) for both farm and companion animals while cruelty to animals is addressed by federal Criminal Code. The federal government has some administrative laws whereas the province has no criminal statutes. Federal administrative law on humane transport of animals was amended in 2020. The previous “Prohibition of Overcrowding” Sec 140 was repealed and replaced by Sec 148 “Overcrowding”. New statutory language did not include a measurable or mathematical threshold of overcrowding. To understand how these narrative prohibitions are enforced, this paper reviews reports of the adjudication of appeals of Administrative Monetary Penalties assigned to violations of the prohibition of overcrowding. This paper reflects upon the history of the regulation of humane transport of livestock in Canada and possible explanation of reluctance for a scientific regulator to incorporate widely accepted numerical standards in promoting a culture of humane transport of livestock.

## 1. Introduction

In 2006, the World Organization for Animal Health (Office International des Epizooties, now the WOAH) recommended animal transport times be kept to a minimum with sufficient space allowance for animals to lie down during transport, with consideration given for climate and ventilation capacity of transport vehicles [1] (now Terrestrial Code, Chapter 7.3). This paper reviews how Canada’s prohibition of overcrowding fails to accomplish this mandate and does not approach the recommendation to assure animals can lie down during transport. Neither the WOAH recommendation nor the Canadian legislation articulate numeric thresholds of limits to crowding.

As a signatory to the OIE/WOAH, Canadian scientists in 2008 believed that global livestock welfare transport standards would soon follow the 2006 international agreement [2]. Almost two decades later, there still is no international consensus of the minimum space that should be allocated to a group of livestock in transport, a question that must have an objective scientific (numerical) solution. The concerned citizen can intuit that an animal needs some minimal space in transit to not experience stress and on longer trips, perhaps, to remain alive.

In Canada, the primary humane transportation of animals’ law is at the national level and has been enforced for decades. This law went under complete revision in 2020. The current humane transportation of animals’ national law paradigm was introduced in 1977 in response to high visibility errors made in the then declining transportation of cattle by rail [3]. The century of livestock transport by rail was replaced by road transport with no cattle transported by rail by the mid-1980s [4]. In regulating rail transport, the regulator addressed a very small client base, primarily the Canadian National Railway and Canadian Pacific Railway. The livestock industry adoption of road transport extended the regulated pool to thousands of regulated entities.

In response to the comprehensive shift in regulatory space, rail car v. truck trailer, the regulator initiated a consultation in 1992 which persisted unabated to a final resolution in 2020, a 28-year consultation period, evidence of a highly politicized arena [3]. Despite the long history and prolonged contention surrounding humane livestock transport, there has never been a scientific measure of overcrowding developed and recognized in law. Numerical thresholds are the norm in administrative law, from food holding temperature to maximal residue limits of chemicals; the specificity of law is necessary to make possible fair enforcement and compliance.

Real world experience of animal handling and transport of meat pigs, for example, provides intuitive conviction that upon occasion a small percentage of slaughter pigs arrive at the abattoir dead or severely disabled due to fatigue-hyperthermia. This outcome could have been avoided by reducing the number of pigs in a compartment so that they could simultaneously lie down and rest (fatigue avoidance). These dead-on-arrival (DOA) and pathologically recumbent (pink and panting) individuals are easily identified. Each individual pig dying from overcrowding induced fatigue-hyperthermia exertional stress experiences very poor animal welfare and has suffered during transit [5]. This is an example of avoidable animal suffering that society agrees should be prohibited.

This paper reviews case adjudication in Canada where transporters are challenging an enforcement action, a monetary penalty, of the offence of livestock overcrowding in transit where there is no science-based measurement, no numerical threshold identified in law as violative. This paper will review how adjudication frames the offences without an objective measurement in humane transport law in Canada. This paper will address the hypothesis that the lack of scientific/numerical threshold for enforcement of the prohibition of overcrowding of livestock in transit is a sub-optimal and a technically correctable regulatory situation. The paper will also postulate as to why the vague legal description of an overcrowding violation was expanded, but not substantively improved, in the 2020 complete revision of the regulation (Table 1).

The remainder of this paper is constructed into four parts; (1) a brief history of the Canadian regulation of livestock in transport; (2) a framework for differentiating the nature of “good” from other types of legislation; (3) a case study of a series of legal decisions on appeal of violation and penalties of overcrowding in transit; and (4) a largely narrative reflection to increase the understanding of common problems in creating and delivery of good animal protection legislation by critique of the Canadian federal law prohibiting overcrowding of livestock in transit with a focus on pigs.

## 2. Brief History of Livestock Transportation Regulation in Canada

British criminal law had been largely exported ver batum for the purposes of governing the British North American Colonies in 1800 [6]. The prevention of Cruelty to Animals Act c. 31, was adopted in British North America in 1857 [7,8], which made it unlawful to bind sheep, calves and pigs (hog-tie) for transport to market, if more than 15 miles distant and to release them from physical restraint within half an hour of arrival at destination. Earlier animal protection provisions in policing acts of Lower-Canada 1845, allowed a Justice of the Peace to place an individual in the Common Gaol for up to a month for brutalizing draught animals in urban settings [9]. Pre-confederation townships, cities, towns and incorporated villages had delegated authority to create by-laws to respond to cruelty to animals and animals running at large [8]; two problems that remain concerns of society.

The British North America Act 1867 (Canadian Constitution) provides the federal government exclusive jurisdiction to legislate criminal offences in Canada, such as cruelty to animals [10]. The BNA 1867 or “confederation” was the administrative union of what is now Ontario, Quebec, Nova Scotia and New Brunswick. At confederation the 1857 colonial act respecting Cruelty to animals c.31 was incorporated as c.27 in Dominion criminal law. In 1875, c.42 An Act to prevent Cruelty to Animals While in Transit by Railway or other means of conveyance within the Dominion of Canada, was promulgated [11]. This act was focused on the prohibition of confining cattle being transported in rail cars, longer than 28 h without feed water and rest (FWR). This Canadian rule mimics the concurrent USA 28 h law that was written in 1873 but not in force until 1909 [12]. In 1887, the Canadian c.27 Animal Cruelty and c.42 Cruelty While in Transit acts were incorporated into a single act c.172 [13]. These acts were further consolidated into the 1892 Criminal Code c.29 with Section 514 Transport of Animals, addressing the FWR requirements [14].

The offence of overcrowding in transit appears in Section 387 of the 1954 Criminal Code [15], a large statute continuously under revision. Overcrowding in transit does not really fit the mens rea, evil intent requirement of criminal law. Overcrowding in commercial livestock transport is more efficiently explained by incompetence, negligence, or error. In 1975, Bill C-28 moved the regulatory oversight for humane transport of livestock from the Criminal Code to the Animal Contagious Diseases Act (ACDA), making animal mistreatment in transit administrative offences rather than the previous criminal offences.

Malicious torture, acts of sadism toward animals and staged animal fighting remain prohibited in the Criminal Code. Bill C-28 was also renamed the ACDA the Animal Diseases Protection Act. This amendment expanded the regulatory powers of a previous exclusively endemic and trans-boundary disease control act [16,17] by providing legislative space to promulgate regulations “to provide for the humane treatment of animals”, Sec 32 [16]. The Animal Disease Protection Act is continued as the current Health of Animals Act. SC 1990, c 21 (statutes of Canada, Chapter 21).

The move from criminal to administrative oversight reflects three major legal shifts in framing: (1) the type of offence calls for penalty not punishment; (2) the type of offence occurs with or without intention (strict liability offence); and (3) criminal law responds to an offence that has already happened. In theory, administrative offences can be minimized with oversight, inspection and penalization. Incarceration for non-compliance with provincial animal protection (non-criminal) law is rare, as in Canadian law, incarceration is punishment. Criminal statutes have no regulations because crimes are prohibited, a type of behaviour where there is no regulation that can make them tolerable and convicted offenders usually are incarcerated. Administrative law bravely attempts to both prevent, and respond, to anti-social behaviour.

Minimizing the pain and suffering of livestock in transit and the sanctioning of those responsible for avoidable animal suffering is largely the domain of the national veterinary infrastructure. In Canada, millions of animals move inter-provincially and internationally with no pre movement or roadside inspection for compliance. Federal inspectors are present to inspect for compliance at the live animal receiving dock of federally inspected slaughter facilities. Over 95% of animals slaughtered for food in Canada are slaughtered at federal plants, producing products which are then eligible for interprovincial and international trade [18]. Part XII, of Health of Animals Regulations, CRC, c 296, (Health of Animals Act), Transport of Animals is enforced by inspectors employed by the Canadian Food Inspection Agency (CFIA) who also provide the humane slaughter and sanitary inspection services at federally registered abattoirs.

Law enabling the identification and enforcement of animal protection is rare at the national level in Canada, a country with dual federalism [19]. The Canadian federal government has some features of the administrative state with the creation of empowered agencies, related to health, food safety and environmental protection, but most limits of individual freedom in Canada are created directly by provincial legislation. Under the Canadian Constitution, the provinces have authority and obligation to fund and operate the Courts and Judicial institutions. The Criminal Code, a national law with a small section re Animal Cruelty, is without an administrative agency, and is supported by uniformed police services. Discovery and prosecution of non-criminal animal law offences remains a local matter under the provincial infrastructure officers, courts and inspectorate functions.

Under dual federalism, a problem of justice emerges in treating all malingering but noncriminal citizens the same, when supporting federal administrative law such as international border control and in this case, the interprovincial movement of livestock. Justice requires that a prohibited food smuggling offence at an international airport in Québec is penalized equally with the same offence identified at a USA-Canada land crossing in British Columbia. The Canadian administrative state addressed this problem by creation of the Canadian Border Services Agency (CBSA). Effective federal policing of noncriminal offences has a problem of accessing provincial justice infrastructure for prosecution and sanctioning [20].

Each province empowers uniformed police and other individuals to act as agents of the justice system to initiate prosecutions. Canada like other British Common Law countries empower police to issue a ticket as a prosecution option for minor common offences. The receiver of the ticket can pay the penalty and decline the right to judicial review. A common choice is to present to a justice, plead guilty, and request reduction in the monetary penalty which is not based on ability to pay as are day fines in other jurisdictions [21]. The Common Offence Notice (CON) system, also known as ticketing, is housekeeping regulatory work where the uniformed officer is empowered to decide the nature of an offence is balanced with the set fine, give a ticket, or whether justice would be better served by requiring the offender to appear before a judge, the ‘Summary Conviction’ process (in Canada).

A provincial street-level [22] animal protection officer, delivering inspection and enforcement of a provincial act, may initiate prosecution by either the CON process (ticket) or initiate a prosecution via the summary conviction process. The summary conviction process is described in the Criminal Code and assures protection of the rights of the accused. Summary conviction process is mirrored in provincial offences acts and is blue collar enforcement work [23]. In proceeding with a CON there is no requirement for the officer to consult with the Crown prosecutor and no information is made accessible to public review, limiting transparency.

The summary conviction system as a process is harmonized across provinces and provincial offences. There is also a more formal indictment process for serious Criminal Code offences such as violent crime, e.g., assault, murder. Criminal Code animal offences such as cruelty to animals or criminal neglect follow the summary conviction process.

The provincial offence summary conviction process and Criminal Code summary conviction process are identical and compete for the resources of Crown prosecutors and the resources of the provincial court system. Summary conviction is used for offences where there is no set fine (complicated, or contestable offences), or where there is a set fine but the details of the incident call for a more significant penalty. In resource demand, a provincial summary conviction charge of starving your livestock requires the same process as a criminal code of animal cruelty without the requirement of proving mens rea.

The summary conviction process is straightforward but complex and burdensome, as it should be, to prevent frivolous police actions. Summary conviction requires enforcement officers to gather detailed information to be shared with Crown prosecutors. Independent Crown prosecutors review the evidence provided and the draft charges, to determine whether any and which charges will be laid. When charges are approved, enforcement officers must present the approved information listing the charges and swear before a Justice as to the veracity of the information. The provincial justice witnesses the officers’ signature, endorses the document and initiates court proceedings. Enforcement officers also prepare summons to be signed by the justice, ordering the accused to appear in court. The officer must also serve the summons to appear on the accused. If the accused opts for a trial by pleading not guilty at first appearance, enforcement officers must provide the required information and assistance to the Crown, with full disclosure to the defence in addition to being available to serve as the primary witness in court.

Enforcement officers must have a significant level of personal commitment to drive the summary conviction process and significant autonomy as their “line manager” (a term sacred in the civil service) is not the Crown prosecutor. The line manager, in the pursuit of justice, must shield enforcement officers from departmental political pressure, so that a farmer residing in the political riding of the Minister of Agriculture is treated the same as farmers not in the Ministers riding. This risk of regulatory capture and politicizing of justice is a frequent criticism where animal protection officers are employed by departments of agriculture [24].

To adapt to the needs of the expanding administrative (non-criminal) federal law, in food safety, environment and agriculture, Agriculture Canada (including CFIA) developed an Administrative Monetary Penalty system (AMPS), [Agriculture and Agri-Food Administrative Monetary Penalties Regulations, SOR/2000-187 [25], (Agriculture and Agri-Food Administrative Monetary Penalties Act SC 1995, c 40)]. The AMPS mimics to some extent the British Common Law summary conviction process, but, outside the provincial court institutions.

In the live receiving at a federal slaughterhouse there is the opportunity to inspect for compliance with the federal humane transportation regulations and discover non-compliance. Inspection for compliance is an inspection where the officer does not expect to find an offence, the discovery of an offence triggers an investigation, which under normal law recruits the human rights protection for the accused. If there are signs of overcrowding at inspection the AMPS process is triggered and the CFIA inspector collects data to support a decision to penalize which remains an inspection for compliance. The decision to assign an AMP results from an investigational review of the information provided in the inspection for compliance. A written Notice of Violation (NOV) is issued to the accused by mail with options for payment. Unlike the CON process, this decision is not made by the primary inspector at the time of discovery, but by a different functionary further up the hierarchy. If the management decision is to penalize, a monetary penalty is calculated using a formula. The accused is notified in writing (NOV) and given the opportunity to agree to the decision and have the fine reduced by 50% by prompt payment. Failure to pay is referred to the Canadian Agricultural Review Tribunal as an appeal.

The AMPS process allows Administrative Agencies to penalize offenders with a monetary penalty significantly larger than the provincial ticketing process allows, but avoids compulsory oversight provided by, review by Crown prosecutors inherent in summary conviction. The summary conviction process by design protects the civil rights and freedoms of the accused as a fundamental requirement of justice and fairness (natural justice). In the arena of federal animal welfare, penalties awarded by the Canadian Food Inspection Agency (CFIA), can be appealed to the Canadian Agriculture Review Tribunal (CART), a legal “court like” organ created by the Act (SC 1995, c 40). Decisions of the CART are appealable to the Federal Court, which is consistent within the framework of British Common Law and natural justice, all convictions have a right of application for appeal.

## 3. Good Law

In common understanding, a “good” law, articulating a social policy or priority, has the intended purpose to bring about behavioural change in the group of people targeted, resulting in a more just society. Legislatures empower regulators to intervene where there is a public good to achieve and where the individual may have cause to do otherwise. A quality assurance programme for the drafting of regulations has emerged in British Common Law, for this discussion, the “Lon L. Fuller Standard” (LLF-8) [26,27]. The LLF-8 does not address the moral value of any legal instrument, only the inherent quality principles that a law or regulation should conform to. The LLF-8 will be used as the quality standard for this discussion. The LLF-8 is not the only quality construct for evaluating just laws; a European variant holds to four critical dimensions; legal certainty, prevention of misuse of powers, equality before the law, and access to justice [28] which does not in any way conflict with the LLF-8, Table 2.

The rule “it is an offence to exceed the posted speed limit” meets the Table 2 quality standard. Speed limits, a socially beneficial proscription, restricting the freedom of action of the individual, is applied to all drivers of vehicles (general), widely promulgated and visible by road signage, directing current and future driver’s behaviour. Speed limits are consistent with all other road safety initiatives, compliance accomplished by decreasing foot pressure on the vehicle throttle control, remaining constant in principle even in school zones and similar special risk zones. The exceptional clarity of this law makes enforcement extremely easy, because an infraction can be objectively and legitimately measured by any one of several technologies including photo-radar systems. It is a scalable, bright line rule, while remaining subordinate to officer discretion, as a serious violation may trigger any process from ticket to criminal indictment. In addition, the licenced driver, the enforcement officer and the courts all see this issue with the same perspective. A good law, it provides a clear method of measuring human behaviour against prohibited behaviour even in the absence of a negative outcome and can ratchet up penalties in proportion to offence severity regardless of specific outcome of the misbehaviour.

Fuller [26] describes law as the “enterprise” of creating and maintaining a functioning society. There is a theory in the development of law that any and all statute development responds to two driving concepts, in varying proportion. The more intuitive driver is identified as prospective–substantive, where the drafters of the law want the machinery of justice to use the law to result in a change in human behaviour increasing societal wellness. The less intuitive driver is identified as political–strategic, where the law is symbolic and does not precipitate changes in human behaviour [29,30]. Symbolic law can be a conscious deception of the public, especially the groups organized and lobbying for the regulation [27]. Law without the promise of enforcement is effectively no law at all, a non-law [26].

There is a rival school of thought that is less critical of symbolic law. This view rests on the ability of law to have norm establishing functions. Law is expressive and communicates a meaning to society [31], a charter of human rights is an example. It is also argued that symbolic law can have a placebo effect in intentionally manipulating public perception to decrease the perception of risk and increase the belief in the law’s effectiveness without a real change in the objective world. An example is the enhanced visibility of airport security post 911, where the increased visibility of airport security did nothing to decrease the real risk of terrorism nor make more effective crime prevention but facilitates the traveller having less anxiety [32]. Other common populist examples include the belief that “tough on crime” laws change human behaviour to make for safer streets [33] and that sex offender registration is protective, because it alters the individuals risk of reoffending. The belief in a positive outcome of these law/policies has no basis in fact [34,35].

Primarily political–strategic laws in animal protection may be common, resulting in no significant real increased protection of animals or measurable relation to offences and prosecution [36]. One can imagine an animal advocacy group campaigning for a particular legal goal such as recognition of animal sentience [37,38] which in the proscriptive-substantive paradigm has no significant impact on the legislative enterprise [39]. In the successful promulgation of ineffective legislation in animal protection, the lobby interests can claim a win (new Law is created) and increase their donations, and the government can claim a win for championing a moral virtue at no real cost to government; even Machiavellian animal use interests that normally oppose methods of production legislation may support a symbolic law.

## 4. Soft Law

“Soft law” in its simplest form consists of widely accepted norms that have no legal basis. It is a measure of rule by administerial discretion as opposed to court and justice rule [40,41]. No real consensus of descriptive criteria for soft law has yet emerged [28] although it has been previously identified in Canada [42]. Hard law includes statutes, regulations and by-laws issued by regulators of professions and municipal authorities, as these powers are delegated by hard law statutes and articulate a penalty process. Soft law includes just about all the other instruments that are issued by the executive to guide internal decision making, or legitimized by being used by the regulated party, including guidance documents, guidelines, codes, manuals, circulars, directives, bulletins, and other forms [43]. They are usually documents authored by the regulatory authority for use of the regulatory authority and those regulated.

The academic definitions of soft law include two types of characteristics. Firstly, soft law is somewhat defined by what it is not, as some essential elements are missing for soft law to be considered (proper) hard law; however, the close resemblance with hard law is what makes soft law differ from non-law. Rather than cross interrogating the wording, soft law is further defined by looking at the function it performs. Soft law may anticipate hard law (pre-law, as in the evolution of tobacco use restrictions in public), follow the adoption of hard law (post-law interpretive guidelines), or be considered as a substitute to hard law (para-law). In this paper, the Canadian Agriculture and Agri-Food Canada 2001 publication of the recommended Code of Practice for the care and handling of farm animals: Transportation [44] is an exemplar of soft law in Canadian livestock regulation. This document has comprehensive livestock transport maximal crowding limits (Density Charts pp. 36–44). Although claiming to be non-law this unchallenged an unimproved document is featured prominently in defending and prosecuting incidents of livestock loss in transit and may be functioning as para-law, see part 7 below. This application of soft law differentiates the Transport Code from the dozen or so other species specific National Farm Animal Care “care and handling” codes [45].

## 5. Malleability of Common Law

One of the characteristics of British Common Law is that by design, it follows an evolutionary pattern of continual improvement. As decisions made in specific cases are decided and appealed, they are reviewed in the context of current legal and social norms and become precedents for decisions in future cases. In addition, the law is delivered in the field by policing functionaries with a high degree of knowledge and discretion [46], which anticipates real world variability and smooths jagged law to make it fit for purpose. Although still recognizable, law is often applied in significantly different form than the authors intended [22]. Police or inspector discretion is fundamental to the modern justice creating enterprise [47].

There is no public information to indicate what proportion of NOVs are appealed to the Canada Agriculture Review Tribunal (CART). The 50% reduction in penalty for early payment is a significant design issue to disincentivize appeal. CART decisions are seldom appealed to the Federal Court. A review of NOV for “undue suffering” during transport between 2000 and 2019 identified 159 CART decisions and only 3 related Federal Court of Appeal decisions [48]. Appeals are expensive, success unlikely and out of pocket cost of winning will probably exceed the other choices. The improvement in common law, however, is dependent on appeals and all appeals have public utility. When administrative power is reviewed by the court of appeals, it can effectively modify the law and change significantly how the administrator functions in the future.

In 2009 a CART ruling related to a NOV of causation of unnecessary suffering in transportation of a severely lame pig, was appealed to federal Court (Doyon v. Canada (Attorney General), 2009 FCA 152) [49]. The appellate judge ruled in favour of CART but had further words regarding the administrative monetary penalty system. The Appellate Judge referred to the system as draconian and highly punitive, where in Canadian legal constitutional norms administrative law is prohibited from being punitive. The AMPS punishes diligent individuals, even if they took every reasonable precaution to prevent the commission of the alleged violation. The Act denies individuals who committed a violation the right to make a mistake, even if the mistake could have been made by a reasonable person in the same circumstances. The AMPS encourages the innocent to assume guilt, by granting a 50% discount on the penalty if the penalty is paid quickly. The Judge indicated, at paragraph [28]:

“In short, the Administrative Monetary Penalty System has imported the most punitive elements of penal law while taking care to exclude useful defences and reduce the prosecutor’s burden of proof. Absolute liability, arising from an actus reus which the prosecutor does not have to prove beyond a reasonable doubt, leaves the person who commits a violation very few means of exculpating him- or herself.”

He went on to admonish the administration of the AMPS: at paragraph [29].

“Therefore, the decision-maker must be circumspect in managing and analyzing the evidence and in analyzing the essential elements of the violation and the causal link. This circumspection must be reflected in the decision-maker’s reasons for decision, which must rely on evidence based on facts and not mere conjecture, let alone speculation, hunches, impressions or hearsay.”

## 6. Methods

The website of the Canadian Agricultural Review Tribunal [50] was searched on using the word “overcrowd*” and the Boolean full text search engine provided by the website, with the time bracket 1 August 2000 to 14 August 2025. The search retrieved 35 results which were downloaded and individually reviewed. Decisions were removed from further consideration where the offence being appealed was not overcrowding but the word overcrowding or overcrowded appeared in the decision. Eight excluded cases were of transporting an unfit animal; inadequate ventilation, exposure to weather, and unfit container (five cases) and there were two written orders unrelated to the appeal process, removing 15 cases.

In the 21 years between 2004 and 2024 there were 20 decisions available for review. The decisions were reviewed by careful reading and compared by length (number of words), whether or not they referred to the 2001 Transportation Code [44], whether the Chairperson used mathematical reasoning in coming to a decision and documented that reasoning in the decision, and the identification of chairperson deciding the file, Table 3.

## 7. Results

**Figure 1:**
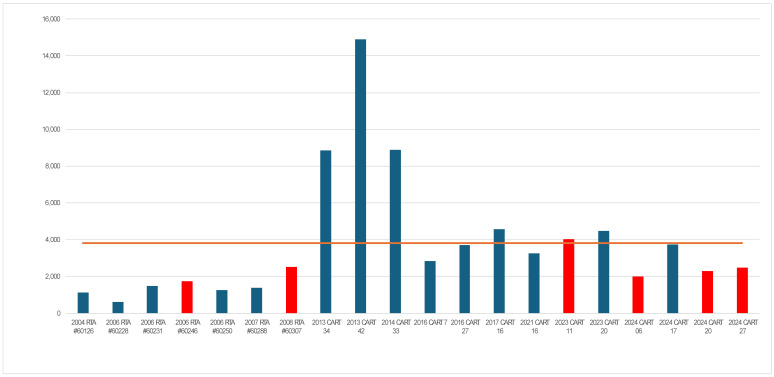
Number of words (*Y*-axis) in each decision (*X*-axis), red line average word count. **The X-axis legend corresponds to the legal case number assigned by the CART appeal process.** Of the three apparent outliers in 2013 and 2014, two were written by DB and 2013 CART 42 was the only file authored by BLR. Bars in red indicate the six cases where the notice of violation and monetary penalty were quashed on appeal. Obscured in the image is the 5-year hiatus from 2008 to 2013 when there were no appeals of violations related to overcrowding.

The 20 files of the species consisted of 1 incident of sheep, 3 cattle, 5 chicken, and 11 pig. The CART annual report of outcome in the year 2023–2024 indicates the Tribunal upheld the violation in 13 files and quashed the notice of violation in 1 (7%) [51]. In this 20-year series of adjudicating overcrowding, 30% (6 of 20) were quashed, 3 pig and one each of cattle, chicken and sheep (Table 3, Figure 1). There were 8 authors (Chairperson) creating the final reports with most having 2 or fewer files with the exception of TSP with 7 decisions from 2004 to 2008 and DB with 5 from 2013 to 2017. A priori, number of words in a decision was considered a proxy for the risk of decisions becoming more litigious over time (Figure 1). Word numbers were counted by exporting the pdf files into MS Word and using the word count function. This theory was erroneous as decisions had two parts: (1) Was the offence substantiated? (2) Was the penalty calculated properly? Cases that were quashed did not have text related to confirmation of appropriate penalty.

The decisions were read closely in an attempt to identify common features and crucial decisions that have potential to act as precedent in future rulings. The 2001 Transport Code of Practice, [44] although consistently introduced in appeals as non-binding, was referred to in all decisions with the exception of 2016 CART 07, a broiler chicken violation where only one cage was overcrowded. The Transport Code sets limits of maximum pressure in units of mass/area; however, there was no numerical information cited in 9 of 20 decisions (Table 3). The value of science, taking measurements in the real world was challenged in Wendzina, 2007 RTA #60228 (paragraphs not numbered), the Tribunal found:

“The determination as to whether the animals were crowded during transport to such an extent as to be likely to cause injury or undue suffering is not a simple matter of applying the actual weights of the animals and the dimensions of the trailer to the loading density chart (page 4, first paragraph, Wendzina decision)”.

And

“In determining whether there is overloading to such an extent as to be likely to cause injury or undue suffering, the type, age and condition of the animals at the time of loading, the number of walls in the trailer, the type and extent of floor covering in the trailer and the weather conditions at the time of loading and during transport are all significant factors to be weighed (page 4, third paragraph, Wendzina decision)”.

The Wendzina, 2007 ruling indicates that “overcrowding” in this legal application, in the absence of a specific numerical rule, is somewhat outside of the realm of science and enters the realm of legal and law enforcement judgement. Overcrowding for the practice of the Tribunal is unknown until the Tribunal rules and it becomes a situationally dependent fact. The creation of fact, transformation objective science, the use of measurement in the real world, to a judicial decision was, confirmed by Para 50 of Finley, 2013 CART 42 even in the presence of objective measurement:

“The Tribunal finds that the Agency has established, on the balance of probabilities, that two of the three dead hogs were in compartments of the transport that were overcrowded, based on national code-referenced calculations, considered to provide indica of overcrowding. Overcrowding remains a question of fact, to which various codes or standards may be referred to in support, but which ultimately becomes a determination based on the particular circumstances”.

In this ruling, despite using the soft law standard in making this determination, the Tribunal clearly retains the future option of disregarding the standard under other circumstances. This example suggests reflecting science in legal rulings is difficult if the process values legal rhetoric over repeatable real world measurement.

## 8. Discussion

This paper reflects on one small institutional arrangement to address a specific cause of avoidable animal suffering in transport, that caused by overcrowding. The behaviour and effectiveness of any enforcement arrangement are difficult areas to critique as review of substantiated welfare violations are rare. Occasionally animal protection investigation records are open to academic review [52].

Overall, from the Tribunal process of review of livestock overcrowding in transport there is limited evidence to believe that the Doyon decision, (at [28]), had a significant or lasting impact on the practice of the Tribunal in reviewing appeals, that of recognition of the significant power imbalance. There is an apparently higher probability of winning an overcrowding appeal than other violations reviewed by CART; 6/20 v. 1/14 [51].

LL Fuller describes law as the “enterprise” of subjecting human conduct to the governance of rules and describes the successful product of science as the ability to predict and control future events. There is a real-world scientific hypothesis of these conditions of maximal safe overcrowding in the units of mass/area. Eschewing the option of including by reference, the Transport Code of Practice and numerical limits seems like an inefficient regulatory choice especially as standards of space allowance for animals by air opts for inclusion by reference.

In the 2020 amendment opportunity, the regulator removed the definitional future looking offence clause, “likely to cause injury or undue suffering”, with focus on the problematic “undue suffering”. The updated Section 148(1) prohibits “loading in a manner that would result in the conveyance or container becoming overcrowded,” which is a weaker future looking rule, necessary as causation is required for culpability and science is firmly focused on identifying ways of predicting the future to avoid things such as undue suffering (48). Future looking offences are created by 148(1)(b) likely to suffer and (c) likely to sustain an injury or die.

In comparing the historic and current overcrowding rules to the LLF-8, does an offence of failure to predict the future ask the impossible of the regulated? The enterprise of science is to understand the nature of reality so as to be able to avoid negative outcomes in the future. In this circular thought, rule 148(1) is theoretically made possible by science, but only if you recruit the wealth of objective knowledge and scientific theory that is open to empirical test and falsification [53].

In a rational prospective science hypothesis, for species that prefer to stand during transportation such as loose horses, there is some level of crowding, if exceeded, a recumbent horse would be unable to regain footing as the remaining horses would close over and prevent efforts of the downer to rise. Similarly, in species that are easily exhausted and prefer to lie down; at some level of crowding, all pigs in a group would be unable to simultaneously lie down without piling on peers. Definition of unlawful overcrowding is 148(1)(a) the animal cannot maintain its preferred position or adjust its body position in order to protect itself from injuries or avoid being crushed or trampled. This description of an offence requires the inspector to make a judgement on the preferences of the group of animals(s) and visible evidence of self-protection. Animal preference is an active and contested field of animal behaviour study, used here as a legally enforceable standard of evidence.

The demands of clarity with what the law prohibits or requires are clearly not met by the current rule. Prohibition of overcrowding with determination based on the particular circumstances cannot be reconciled with the requirement of clarity. The retention of the “likely to” clause in 148(1)(b) and (c) also requires the regulated to see into the future without any officially objective legal guidance. In this application of legal compulsion, is the rule actually compliable? It appears the law is asking the impossible of the regulated. In 2024 CART 27, this future looking aspect of the regulation was directly confronted and the decision read;

“The Agency has not convinced me on a balance of probabilities that it was likely that an animal would suffer, sustain an injury or die due to the number of animals in the container” [Para 34].

In this series of appeals there is also evidence of lack of congruence between what written rule declares and how officials enforce the rule. In 2023 CART 20 at para [31]:

“While I am sympathetic to Brussels’ concern about the apparent arbitrariness of the Agency’s decision to only investigate when more than three hogs arrive dead in a single load, the Tribunal has no mandate to specify that all breaches of the regulations be enforced.”

In 2024 CART 06 at para [39], a similar situation was documented. Inspector Amanda Murphy testified that the regulations in question are “outcome based”. She stated, repeatedly, that the Respondent will not pursue enforcement if an otherwise overcrowded trailer does not show any negative outcomes (like hyperthermia). Inspector Murphy’s testimony is consistent with Section 15.2 of the Respondent’s Interpretive Guidance document.

In operational reality, the enforcement of overcrowding is only initiated where a significant number of animals die. The arbitrary threshold, the size of the pile of dead pigs, communicates to the regulated that, it is death due to overcrowding that is prohibited not overcrowding per se. There is no data available of the frequency of overcrowding on short hauls where stress is not of sufficient temporal duration to result in death of pigs.

The Veterinarian in Charge of the federal slaughter facility is the unfortunate street-level bureaucrat tasked with balancing the resources to inspect and document animal welfare and the resources to inspect and document food safety requirements. On shifts with a staff shortage, it is likely data collection at live receiving may suffer. In Canada, there is no roadside monitoring of livestock compartment floor pressure nor monitoring at assembly points, auction markets or provincial and uninspected abattoirs. The popular practice of comparative evaluation of animal protection of jurisdictions based on statute review only [54] is misleading at best, as it ignores the reality and constraints of street-level enforcement.

CFIA self identifies as Canada’s largest science based regulator [55] yet, eschews numerical thresholds for the definition of an offence of overcrowding in inspection for compliance, yet rely on soft law standards [44] in further adjudication. CFIA was the primary sponsor of the 2001 Transport Code which remains available in electronic format and classified as “archived” under the current management of the National Farm Animal Care Council. There is no intention of reviewing this code in the strategic planning of the Council. The Council considers the CFIA interpretive guidance documents [56] to have replaced a great deal of the Transport Code with the exception of the floor pressure graphs. The CFIA interpretive guidance documents do not provide any numerical violative limits of the mass of live animal per floor area of compartment [56].

The guidance on identifying individual animals unfit to travel Sec 139, [56] is quite extensive, while there are no numerical recommendations on overcrowding. A reasonable explanation for this is that current overcrowding provisions are symbolic legislation, intentionally worded for political not substantive ends. In most critical speculation there may have been no legislative intent to enforce the overcrowding provisions evidenced by making wording very difficult to comply with and difficult to prosecute, in comparison with a numerical threshold. Without being in the room while the decisions are made, it is impossible to judge the intent of the legislature or their advisors. An efficient explanation is that sometime in the 30-year negotiations up to the recent revisions of the regulation it was decided that the issue of overcrowding was unlikely to be successfully negotiated with industry; any possible scientific and easily enforceable definition of overcrowding was traded for other concessions in the regulatory proposal.

The limitations of this study are significant. Public information is only available for the incidents where the accused appealed the penalty. In 2014 CART 33, Western Commercial Carriers Ltd. v. Canada (CFIA), there were 30 DOA pigs on arrival and one additional pig distressed. The appellant believed that a penalty was not justified in the face of 31 dead pigs. This example triggers two major concerns. How frequently do 31 pigs die on a load of 270 pigs and the accused promptly pays the reduced 50% of the calculated penalty and the incident is closed. In prompt penalty payment no public information ever released, and after 5 years the record is expunged. In the structure of the AMPS process, how many pigs does one have to kill to risk being brought before a judge in regular court? The apparent answer is, there is no Health of Animals Regulation offence, so vile that it requires escalation into the normal justice system to achieve justice; the mechanism does not exist.

This second concern can be applied in normal law enforcement. Under The Provincial Offences Act (Manitoba) [57] there are set fines addressing common failure to provide the necessities of life, Sec. 2(1) of the Animal Care Act (Manitoba). For the first offence of failing to provide food and water for a single animal the fine is CAD 298.00 per animal for a maximum of two animals; if your first offence concerns three or more animals or it is a second offence the fine is escalated to CAD 672.00 for animals starved but alive. If, however, the animal dies from neglecting to provide feed or water the fine is CAD 1296.00. The provincial statute articulates suffering leading to death attracts greater financial sanction than suffering survived.

In a Provincial case of severe animal suffering, the enforcement officer has the responsibility to advance the appropriate legal sanction. There is incentive to issue a set fine where available, considering the CON process immediately ends the time commitment of the officer and carries a low risk of review. The summary conviction process on the other hand is a commitment to hundreds of hours of subsequent work. The federal AMP system greatly reduces the risks of public criticism on internal decisions made by government actors tasked with the administration of justice. Animal protection delivered by provincial agriculture employees can mimic this protection of offender privacy by choosing the ticket option over summary conviction for all incidents.

The delivery of justice for the more than human society can be challenging. The question remains of how many animals does a person need to cause to die, to earn an appearance before a judge to answer for their behaviour? In Manitoba, where overwinter cattle starvation is common [58] the set fine standard is silent on more than two animal deaths. This suggests that three animal deaths from starvation would or should trigger a proceeding by summary conviction. Previously in Manitoba in livestock enforcement a 10% death loss from starvation was the criteria to trigger resource commitment to pursue summary conviction [58].

The internal workings of the government, where this paper attempts to peer, are opaque to the common citizen. No outsider is privy to the intent in the creation stages in the regulatory process. The data source used was limited to the transparency window made available by the current Tribunal publication policy. There is no public information to compare the proportion of animal welfare violations in the overall workings of the Tribunal or the fraction of welfare violations that are issued due to overcrowding. There is also no data publicly available to document the proportion of violators that pay half the penalty, regardless of guilt, motivated not by a sense of remorse, but to avoid the cost and inconvenience of the appeal process. In addition to the arbitrary investigation threshold of some number of dead pigs on arrival, of 3(2024, CART 06) there is no assurance that this internal policy is regularly enforced at street-level or how the number of violations would increase with a change in the dead pig pile trigger for investigation. This review would suggest that overcrowding is far more common than is indicated by the evidence available to the public by the Tribunal transparency policy.

A final concern in the decision to adopt an AMPS to address non-compliance is the barriers to escalating the most serious offences to achieve proportional sanction and justice. Most human negligence is not criminal; however, human behaviour includes action that is criminal [59]. This directed reading revealed two incidents were the death loss in transit exceeded 10% of the number of healthy animals loaded, and the offender appealed the Notice of Violation; appeal 2004 RTA60126, 12/115 DOA pigs, and 2014 CART 33, 30/270 pigs. In a related review of CART appeals of the charge of “undue suffering” the author indicated “there are unsettling revelations about what industry and government actors considered to be **normal** (and therefore tolerable suffering)”, emphasis in original [48].

## 9. Conclusions

This project was curiosity-driven from the perspective of a previous street-level inspector with 15 years of wrangling animal neglect/abuse cases through the summary convictions process of a Canadian provincial court. Describing the frame within which the federal prohibition of overcrowding in transport is enforced required an introductory description of how general human responsibility for the care of non-human is assured through the court system in Canada. This resulted in a comprehensive narrative of the Canadian animal protection legal system, which has not been described elsewhere. A review of the appeal of federal prohibition of overcrowding in transport revealed structural weaknesses that centre on (1) the lack of consideration for animal welfare in the transportation of livestock and (2) the lack of real transparency in documenting animal protection delivery system.

Future research into this question should use the paradigm of “regulatory failure”, originating in the economics literature, where there is assumed financial interest that benefits from the peculiar dysfunctional arrangement [60]. This document is a strong example of interrogating animal welfare inspection and enforcement in industrial livestock production is a multidisciplinary project.

## Figures and Tables

**Table 1:** Change in the statutory wording of the National Prohibition of Overcrowding.

Repealed		Replaced 22 April 2020
**Prohibition of Overcrowding** 140 (1) No person shall load or cause to be loaded any animal in any railway car, motor vehicle, aircraft, vessel, crate or container if, by so loading, that railway car, motor vehicle, aircraft, vessel, crate or container is crowded to such an extent as to be likely to cause injury or undue suffering to any animal therein. (2) No person shall transport or cause to be transported any animal in any railway car, motor vehicle, aircraft, vessel, crate or container that is crowded to such an extent as to be likely to cause injury or undue suffering to any animal therein.		**Overcrowding** 148 (1) No person shall load an animal, or cause one to be loaded, in a conveyance or container, other than a container that is used to transport an animal in an aircraft, in a manner that would result in the conveyance or container becoming overcrowded, or transport or confine an animal in a conveyance or container, or cause one to be transported or confined, in a conveyance or container that is overcrowded. (2) For the purposes of subsection (1), overcrowding occurs when, due to the number of animals in the container or conveyance, (a) the animal cannot maintain its preferred position or adjust its body position in order to protect itself from injuries or avoid being crushed or trampled; (b) the animal is likely to develop a pathological condition such as hyperthermia, hypothermia or frostbite; or (c) the animal is likely to suffer, sustain an injury or die. 148.1 No person shall transport an animal by air, or cause one to be transported by air, unless it is transported in a container that meets the stocking density guidelines that are set out in the Live Animals Regulations, 44th edition, published by the International Air Transport Association, as amended from time to time.

Note: For animal transport by air, the CFIA can enforce the IATA standards as they are incorporated by reference into the regulation.

**Table 2:** LLF-8: eight Fuller characteristics of high-quality law and competent lawmaking.

general	High specificity of the rules prohibiting or permitting behaviour of certain kinds
widely promulgated	Publicly accessible rules as publicity of laws ensure citizens know what the law requires
prospective	Specifying how individuals ought to behave in the future rather than prohibiting or sanctioning behaviour that occurred in the past
clear	Citizens should be able to identify what the laws prohibit, permit, or require
non-contradictory	One law cannot prohibit what another law permits
compliable	Must not ask the impossible of the regulated
constant	The demands laws make on citizens should remain relatively constant, laws should not change frequently
congruence	There should be congruence between what written statute declares and how officials enforce those statutes. Congruence requires lawmakers to pass only laws that will be enforced and requires officials to enforce no more than is required by the laws.

**Table 3:** Summary of Tribunal decision.

Ref.	Reference	Word Count	Code	Math	Chair	Description	Sect.	Case Turns on….	Outcome
2004 RTA #60126	F. Ménard Inc. v. Canada (CFIA)	1137	Y	N	TSB	4 August, daily high of 29 °C and high humidity, the applicant loaded 115 hogs from several farms and transported them to the slaughterhouse. At the time of unloading 12 hogs were dead and the remainder of the lot were demonstrating symptoms related to heat stress.	140(2)	The transporter did not decrease the floor pressure to the extent recommended by the Transport Code of Practice.	Notice upheld: The applicant committed the violation and is liable for payment of the penalty in the amount of CAN 2000.00 to the respondent within 30 days
2006 RTA #60228	Transport Giannone-Garceau Inc. v. Canada (CFIA)	624	Y	Y	TSB	The applicant transported a load of chickens from Woodstock, ON to Drummondville, Quebec, (784 km) on the evening of 4 October and morning of 5 October. Eighty cages contained eight chickens each and five hundred and fifty-six cages contained seven chickens each. The chickens had an average live weight of 3.86 kg. A large number of dead birds were found in cages located in the centre of the trailer more than 10%.	140(2)	The Transport Code recommends a maximum live weight loading density in cold weather of 63 kg/m^2^, equates to 7.4 chickens per cage in this case.	Notice upheld: The deaths (and undue suffering) of the chickens was caused by overcrowding in the circumstances and accordingly has established on a balance of probabilities that the applicant committed the violation. Reduced the penalty from USD 4400.00 to CAN 2200.00.
2006 RTA #60231	René Nadeau v. Canada (CFIA)	1497	Y	Y	TSB	1 March loaded 780 crates of 45-day old broiler chickens, to be transported to Grand River Poultry located in Beamsville, Ontario. At slaughter the birds were diverted to a second processor because they were too large for the processor settings. The weather conditions were fair and cool throughout transport, total travel time 26.5 h. The DOA rate was 7.22%, 332 birds were condemned.	140(1)	The recommended Code of Practice for chickens the recommended maximum live weight loading floor pressure for crates in cold weather is 67.9 kgm^−2^, whereas the density in this load was 71.8 kgm^−2^.	Notice upheld: Tribunal determined the applicant committed the violation, by allowing these chickens to be crated and transported in the manner they were. and is liable for payment of the penalty of CAN 2000.00 to be paid within 30 days.
2006 RTA #60246	Curtmar Farms Limited v. Canada (CFIA)	1738	Y	N	TSB	On 6 March, two front compartments contained 12 calves in the upper section and 13 calves in the lower section on an overnight trip from Carbonear to Port aux Basques ND (866 km) took approximately ten to twelve hours and loaded on a ferry to Quebec. Weight of calves recorded but not area of compartment.	140(1)	Insufficient numerical data in the decision to calculate floor pressure.	Quashed: The evidence overcrowding is based almost entirely on the weight of the animals as estimated by the young and inexperienced driver, and which evidence has been put in considerable doubt by the direct evidence of the applicant. Lack of supporting evidence
2006 RTA #60250	Curtis Edwards v. Canada (CFIA)	1266	Y	N	TSB	Loaded 45 cattle (cull dairy cows) at Prince Albert SK on 26 October, and arrived at destination in Calgary AB on 27 October, at noon, to travel 741 km distance. A downer animal was identified at Taber AB 701 km mark. This animal was DOA and 2 further animals were down.	140(2)	The belly of the trailer contained one animal over the maximum recommended limit, and the back compartment contained 2 animals over the maximum recommended limit by reference to the Transport Code.	Notice upheld: The applicant committed the violation and is liable for payment of the penalty in the amount of CAN 2000.00 to the respondent within 30 days.
2007 RTA #60288	Timothy Wendzina v. Canada (CFIA)	1398	Yes	N	TSB	It is undisputed that on 1 March, the applicant transported a load of 38 dairy cattle (Holsteins with one Jersey) and a bull from the farm to XL Beef in Moose Jaw, Saskatchewan, a distance of approximately 250 km. The weather conditions were a temperature of −8 °C, wind at 28 km/hr gusting to 41 km/h, and a windchill of −17 °C. It was overcast with light snow. On arrival there were multiple cows down in the belly of the trailer and ambulatory cattle had to be unloaded over top of one of them. Three animals were unable to stand, were recumbent, non-responsive, wet, covered in excrement, and had to be euthanized.	140(2)	The problem compartment was overcrowded by the Code standards, but dead cows were not weighed, and other numerical facts do not appear in the decision.	Tribunal determined the applicant committed the violation and is liable for payment of a reduced penalty in the amount of CAN 2200.
2008 RTA #60307	Brian’s Poultry Services Ltd. v. Canada (CFIA)	2498	Y	Y	TSB	On 4 October, two loads of chicken; Load A 4532 chickens (80 crates at 8 birds per crate and 556 crates at 7 birds per crate). The average weight per crate was 27.02 kg, 453 dead birds were found (10% of load). Load B 5460 chickens (780 crates at 7 birds per crate). The average weight per cage was 26.88 kg 590 dead birds were found (10.8% of load). Both trucks travelled Woodstock ON to Drummondville PQ, 786 km.	140(1)	BPS presented the Transport Code recommending the maximum live weight loading densities for chickens in crates in cold weather to be 63 kg/m^2^. Load A was 12.1% below the recommended maximum; and load B was 12.5% below the recommended maximum, Crate weight measured floor area of a crate not measured at inspection.	Quashed: The Tribunal indicated that death is evidence of suffering. Tribunal determined the applicant did not commit the violation and is not liable for payment of the penalty. Decision to overturn was based on the reporting of the floor pressure of crates being below the soft law standard.
2013 CART 34	0830079 B.C. Ltd. v. Canada (CFIA)	8864	Y	N	DB	On 25 July, transported 6018 spent laying hens on 2 trailers: 3600 in Trailer A, 84 crates (28 chickens/crate) plus 48 crates (26 chickens/crate) and 2418 in trailer B, 96 crates (26 chickens/crate). At slaughter, Trailer A had 703 DOA and Trailer B had 124 DOA. Size of crate not measured or not recorded in decision.	142(2)	Service contract stipulated 18 birds/crate. Code of Practice recommended maximum was 19 bird/cage for trailer A and 18 bird/cage for trailer B. Actual number of birds loaded was 28 and 26 birds per cage.	Notice upheld: On the balance of probabilities, the accused committed the violation and is liable to pay the respondent, the Canadian Food Inspection Agency, a monetary penalty of CAN 3000 within thirty (30) days (reduced from CAN 6000).
2013 CART 42	Finley Transport Limited v. Canada (CFIA)	14,888	Y	Y	BLR	5 August, afternoon 205 hogs loaded at Petrolia ON to Toronto ON slaughterhouse 278 km, at 30 °C; 3 dead on arrival one live unable to stand, dead in three different compartments. Pigs in respiratory distress, panting with oral froth. Recorded compartment floor space, pig weight not available assumed 230 lb (105 kg). The 6 central compartments in the potbelly area of the trailer were loaded at 242 kgm^−2^.	140(2)	Finley did not adjust the number of pigs/compartments in response to the hot ambient temperatures. Ruled death was from heat exhaustion and death of this type is significantly foreseeable.	Notice upheld: Defence claimed preexisting heart conditions as cause of death. The Tribunal established, on the balance of probabilities, that the three hogs in question died from heat exhaustion due to overcrowding and orders t pay the Agency a monetary penalty of CAN 6000 within thirty (30) days
2014 CART 33	Western Commercial Carriers Ltd. v. Canada (CFIA)	8867	Y	Y	DB	On 1 July the appellant transported 270, 113 kg pigs (best weather capacity of trailer though to be 280 pigs) in the early morning that day in Falher, AB and then driving the to Langley, BC (1266 km). He arrived in Langley very early in the morning of 2 July 2013. At 07:00 that morning, as the pigs were unloaded at the slaughterhouse, 30 pigs were DOA and 1 distressed pig killed at unloading.	140(1)	Did not reduce number loaded to 75% of his best weather load. Eight of the 12 compartments of the truck had dead hogs, with 4 compartments having 5 DOAs, no clustering within a compartment. Average floor pressure across 12 compartments was 251 kgm^−2^ of live pig at loading.	Notice upheld: On a balance of probabilities, the applicant, committed the alleged violation, and orders it to pay to the respondent, the amount of CAN 6000.00 within 30 days.
2016 CART 07	TransVol Ltée v. Canada (Minister of Agriculture and Agri-Food Canada)	2827	N	N	DB	8 and 9 March the appellant a commercial broiler chicken catching company loaded 7340 birds into 734 cages, 10 birds each and 14 additional cages on the truckload left empty. The load arrived at slaughter in slightly more than 1 h. Upon arrival officials found 48 dead chickens and one cage containing 20 chickens with 6 dead. Only the one cage was considered overcrowded.	140(1)	TransVol does not contest the fact that the 20-bird cage was found on a truck, but rather it alleges that the particular cage in question was not filled or loaded onto the truck by its employees. It is impossible to cram 20 chickens weighing 2.37 kg into the type of cage used in this case; and some of the chickens must have been culls and weighed far less than the average 2.37 kg.	Notice upheld: On a balance of probabilities the applicant, TransVol Ltée., committed the alleged violation, and ordered it to pay, the Canadian Food Inspection Agency, a monetary penalty in the amount of CAN 7800.00 within 30 days
2016 CART 27	Transport Robert Laplante & Fils Inc. v. Canada (CFIA)	3696	Y	N	DB	On 17 May, the applicant transported 237 pigs for 3 h from farm to slaughter. Due to a miscalculation, he believed he had only loaded 232 pigs. On arrival 2 pigs were found DOA and 2 further pigs were stressed.	140(2)	No numerical evidence, nor location of dead and compromised pigs was contained in the decision.	Notice upheld: On a balance of probabilities, the applicant, committed the alleged violation, and orders it to pay to the respondent, the amount of CAN 6600.00 within 30 days.
2017 CART 16	Transport Eugène Nadeau Inc. v. Canada (CFIA)	4559	Yes	No	DB	On 14 May, an unusually hot day high 27.7 °C, the applicant transported 180 pigs coming from two barns about 70 km to the abattoir taking 9 h (mechanical failure on road), arriving on 15 May. The loading of the pigs started at 07:00 a.m., and the pigs were finally unloaded at the abattoir at 16:00 p.m. At unloading 10 DOA on top deck, some of the pigs appeared to be in distress.	140(2)	At the time of the incident the trailer compartments were measured and compared to the code maximum. No numerical evidence was recorded in the decision.	Notice upheld: On a balance of probabilities, the applicant, Transport Eugène Nadeau Inc., committed the alleged violation, and orders it to pay to the respondent, the Canadian Food Inspection Agency, a monetary penalty in the amount of CAN 7800 within 30 days
2021 CART 16	C.I. Hishon Transport Inc. v CFIA	3251	Y	Y	LB	On 13 July, 211 hogs weighing 125 kg were loaded into a trailer from three different farms located in relative proximity to each other in western Ontario. The load then made its 13 h journey of about 725 km to the slaughterhouse facility in Saint-Esprit, Quebec. On arrival there were 7 dead and 2 in respiratory distress.	140(2)	Dead and compromised pigs were all confined to the rear compartment loaded at 334 kgm^−2^. With 7 pigs dead the floor pressure was reduced to 267 kgm^−2^ and 2 additional pigs were dying.	Notice upheld: On a balance of probabilities The CFIA has demonstrated a causal link between Hishon Transport and the transport, the crowding, the actual injury and undue suffering confirmed the penalty in the amount of CAN 7800.00.
2023 CART 11	Atkinson v CFIA	4010	Y	Y	MR	Brandon Manitoba 328 sheep to Slaughter in Ontario (no destination reported) three animals with injuries, each in separate compartments: a dead lamb, a downed cull ewe that was subsequently euthanized and a dead newborn lamb.	148(1)	Truckside calculations of floor pressure were presented as evidence. Inspectors were using transport Code of Practice standard, that evidence was not documented in the decision. Dead ewe was old and emaciated.	Quashed—the Agency did not prove on the balance of probabilities overcrowding caused injury or undue suffering to the dead lamb or the downed cull ewe. Overturned on weakness of evidence
2023 CART 20	Brussels Transport Ltd. v CFIA	4482	Y	Y	PLF	Denfield ON to Saint-Espirit PQ 772 kilometres in 8.5 h at 24–30 °C. Of 170, 130 kg market hogs, five were found dead in the trailer in 2 different compartments upon their arrival at the slaughter house; 3 of 24 and 2 of 18. Other animals were observed in respiratory distress upon arrival. This federal slaughter facility documents and investigates loads with 3 or more DOA.	148(1)	Accepted maximum allowable floor pressure in standing room only pigs, best possible weather and trip duration to be 287 kgm^−2^. The code requires reduction in crowding by 25% in hot weather. In Compartment A loaded at 238 kgm^−2^, 3 of 24 died; Compartment B, loaded at 314 kgm^−2^, 2 of 18 died. The weather warranted a 25% reduction in floor pressure as recommended by the Code.	The Notice is upheld. Brussels failed to reduce the density of hogs in two compartments by 25% in response to the hot, humid weather during the hogs’ transport. Maximum loading pressure of market pigs in summer weather (soft law) is 3/4 of 287 kgm^−2^ is 215 kgm^−2^ both compartments were overloaded, penalty of CAN 13,000.00 confirmed.
2024 CART 06	1230890 Ontario Limited v CFIA	1993	Y	Y	EC	Appellant confined 167, 128 kg, market hogs from 3 farms near Kerwood, ON, and transported them to Breslau, ON, 156 km at 27 °C, 1hr 29 min. Dead pigs were identified in 3 compartments	148(1)	Adopted Ontario Pork recommendation that for ambient temperature from 24 °C to 28 °C to reduce load by 15% from the National Code. Tribunal compared the 10 compartments to the standing room only 287 kgm^−2^.	Compartments ranged from 103 to 245 kgm^−2^. The most crowded compartment has reduced floor pressure by 14.6% and contained no dead pigs. Quashed—The Notice and its penalty of CAN 10,000.00 are cancelled.
2024 CART 17	Earl MacDonald and Son Transport Limited v CFIA	3722	Y	Y	GP	On 10 August, 171 pigs were loaded and transported at 26–28 °C (840 km) in a triple axle potbelly trailer from Thamesville ON to Saint-Espirit PQ. In the rear compartment, 3 of 27 pigs loaded were DOA. Some pigs were panting on arrival, but had no other abnormalities (hernia, lameness, etc.) and were placed in a pen to cool off under showers provided for this purpose.	148(1)	Facts: 27 pigs. average 121 kg loaded in a compartment of 12.65 m^2^ a floor pressure of 258.3 kgm^−2^. Accepted (soft law) maximum allowable floor pressure, in standing room only pigs, accepted as be 287 kgm^−2^. The Ontario Pork guide requires reduction by 15% in hot weather, or a maximum floor pressure of 244 kgm^−2^. The compartment was overloaded by 3 pigs.	Notice upheld: Violation occurred at the time of loading and did not require the death of pigs. Confirm the administrative monetary penalty provided for in Notice of Violation in the amount of CAN 10,000.
2024 CART 20	Vernla Livestock Inc. v CFIA	2292	Y	N	PLF	Two incidents where market pigs loaded 205 hogs on the 30 December. 2 pigs stressed and killed on arrival, and 260 hogs on 18 January; 3 hogs DOA, 2 further hogs, were non-ambulatory on arrival and euthanized. First appeal where individual hog weight was estimated by the difference between loaded and empty trailer weight precluding compartment floor pressure calculations.	148(1)	Due to the number of animals in the container, an animal was likely to suffer, sustain an injury or die. This case is based on the likelihood of a future event. No floor pressure information was presented in the decision although CFIA calculations of floor pressure were accepted in evidence.	Quashed—the penalties of CAN 11,000.00 and CAN 13,000.00 as the Agency has failed to prove that Vernla committed the violations of overcrowding. Decision to overturn: Agency did not prove average weight of hogs, floor pressure could not be calculated which is the basis of the offence.
2024 CART 27	1230890 Ontario Limited v CFIA	2487	Y	Y	MR	On 28 January, the Applicant loaded 270, 135 kg, market hogs at a farm and transported them to an abattoir. Eight compartments held 30 hogs each and 2 compartments held 15 hogs. The load arrived with 7 dead hogs and 3 non-ambulatory hogs distributed in 6 compartments. At unloading pigs were panting, an unusual presentation in winter. Weight of pigs was recorded in ruling but area of compartment(s) was not	148(2)	Due to the number of animals in the container, an animal was likely to suffer, sustain an injury or die. This case is based on the likelihood of a future event. It is not “outcome-based” as argued by the Agency. No floor pressure information was presented in the decision although CFIA calculations of floor pressure were accepted in evidence.	Quashed: The Agency has the burden of proof. Quote: “The Agency has not convinced me on a balance of probabilities that it was likely that an animal would suffer, sustain an injury or die due to the number of animals in the container.” Penalty of CAN 13,000.00 was cancelled. Decision to overturn: Rational for overturning the decision is not clear, no clear reason was given for disbelief of overcrowding. Emphasis may have been requirement to predict the future in the clause “likely to suffer”. There was no indication of emphasis in the printed decision.

Note: Accepted soft law maximum allowable floor pressure in standing room only pigs, best possible weather and trip duration 3 h or less to be 287 kgm^−2^. All documents are open access at https://decisions.cart-crac.gc.ca/cart-crac/en/nav.do, accessed on 15 August 2025. Where the word code is used in this table it refers to the 2001 Humane Transport code [44]. Shaded decisions indicate Tribunal overturn of the original Notice of Violation decisions (Table 3). The value of science, taking measurements in the real world was challenged in Wendzina, 2007 RTA #60228 (paragraphs not numbered), the Tribunal found.

## Data Availability

The data used in this manuscript is in the public domain and available from the webpage of The Canadian Agricultural Review Tribunal, https://decisions.cart-crac.gc.ca/cart-crac/en/nav.do, accessed on 1 September 2025.
